# Pituitary adenoma consistency affects postoperative hormone function: a retrospective study

**DOI:** 10.1186/s12902-023-01334-1

**Published:** 2023-04-26

**Authors:** Dario De Alcubierre, Giulia Puliani, Alessia Cozzolino, Valeria Hasenmajer, Marianna Minnetti, Valentina Sada, Valentina Martines, Antonella Zaccagnino, Andrea Gennaro Ruggeri, Riccardo Pofi, Emilia Sbardella, Mary Anna Venneri

**Affiliations:** 1 Department of Experimental Medicine, Sapienza University of Rome, Rome, Italy; 2 Oncological Endocrinology Unit, IRCCS Regina Elena National Cancer Institute, Rome, Italy; 3 Neuroradiology Division, Sapienza University of Rome, Rome, Italy; 4 Department of Neurology and Psychiatry–Neurosurgery Unit, Sapienza University of Rome, Rome, Italy; 5 Oxford Centre for Diabetes, Endocrinology and Metabolism, NIHR Oxford Biomedical Research Centre, University of Oxford, Churchill Hospital, Oxford, UK

**Keywords:** Pituitary adenoma, Hypopituitarism, Tumor consistency, Postoperative pituitary function

## Abstract

**Background:**

Tumor consistency recently emerged as a key factor in surgical planning for pituitary adenomas, but its impact on postoperative endocrine function is still unclear. Our study aimed to evaluate the impact of tumor consistency on the development of postoperative pituitary deficiencies.

**Methods:**

Single-center, retrospective analysis of consecutive pituitary surgeries performed between January 2017 and January 2021 at Policlinico Umberto I in Rome. All patients underwent radiological and biochemical evaluations at baseline, and hormone assessments 3 and 6 months after pituitary surgery. Postoperative MRI studies were used to determine resection rates following surgery. Data on tumor consistency, macroscopic appearance, neurosurgical approach, and intraoperative complications were collected.

**Results:**

Fifty patients [24 women, mean age 57 ± 13 years, median tumor volume 4800 mm^3^ [95% CI 620–8828], were included. Greater tumor volume (χ^2^ = 14.621, *p* = 0.006) and male sex (χ^2^ = 12.178, *p* < 0.001) were associated with worse preoperative endocrine function. All patients underwent transsphenoidal adenomectomy. Fibrous consistency was observed in 10% of patients and was associated with a Ki-67 greater than 3% (χ^2^ = 8.154, *p* = 0.04), greater risk of developing postoperative hormone deficiencies (χ^2^ = 4.485, *p* = 0.05, OR = 8.571; 95% CI: 0.876–83.908), and lower resection rates (χ2 = 8.148, *p* = 0.004; OR 1.385, 95% CI; 1.040–1.844). Similarly, worse resection rates were observed in tumors with suprasellar extension (χ2 = 5.048, *p* = 0.02; OR = 6.000, 95% CI; 1.129–31.880) and CSI (χ2 = 4.000, *p* = 0.04; OR = 3.857, 95% CI; 0.997–14.916).

**Conclusions:**

Tumor consistency might provide useful information about postoperative pituitary function, likely due to its impact on surgical procedures. Further prospective studies with larger cohorts are needed to confirm our preliminary findings.

**Supplementary Information:**

The online version contains supplementary material available at 10.1186/s12902-023-01334-1.

## Background

Pituitary adenomas (PAs) are common intracranial neoplasms, accounting for 15% of brain tumors. In most cases, they are clinically silent, with epidemiology studies reporting a 16% to 20% prevalence of pituitary incidentalomas in the healthy population [1]. Their clinical significance mainly lies in possible autonomous hormone secretion and mass-related complications typically associated with large adenomas, such as headache, visual defects, and hypopituitarism [2].

Surgical resection is the current treatment of choice for large symptomatic tumors as well as for hormone-secreting Pas [3], with the exception of prolactinomas. The primary aims of pituitary surgery are to reduce mass-related effects and normalize hormone secretion [4]. Given the potential damage to the anatomical components of the *sella turcica* (such as the healthy pituitary gland, blood vessels, the visual pathways and the hypothalamus), pituitary surgery bears the risk of potentially serious complications, including cranial nerve defects, iatrogenic hypopituitarism, diabetes insipidus and, more rarely, cerebrospinal fluid leak, bleeding, and infections [5]. However, the advent of increasingly precise and more standardized neurosurgical techniques and improved equipment have drastically reduced the prevalence of surgery-related complications [6].

In relation to postoperative hormone secretion, recent studies have reported that selective endoscopic adenomectomy is often associated with an improvement in previously impaired pituitary function, especially in patients presenting with multiple preoperative hormone deficiencies [7]. However, the endocrine outcomes of pituitary surgery remain largely unpredictable, and the extent and timing of postoperative recovery of secretory function is extremely variable in published series [8, 9].

Several factors have been associated with postoperative hormone dysfunction, including pre- and postoperative hormone levels (in secreting lesions), patient age, tumor volume, and cavernous sinus invasion [10]. However, to our knowledge, the role played by tumor consistency on iatrogenic hypopituitarism following endoscopic transsphenoidal adenomectomy (TSA) is still unclear.

Pituitary adenomas are typically soft, while a firm consistency is only observed in a limited percentage of cases, ranging from 5 to 13% [11]. Tumor consistency has been proven to play a pivotal role in affecting the surgical outcome in pituitary surgery. Soft consistency usually facilitates tumor removal, which can occur through spontaneous detaching during the surgical opening, suction, or curettage. Conversely, a hard texture can greatly impair the surgical resection rate due to the complicated anatomic structure in the *sella turcica* region and the limited operative field of view [12]. Indeed, fibrous (firm) tumors are associated with higher rates of recurrence [13], cavernous sinus invasion, and sellar floor erosion [14], as well as worse surgical outcomes, including lower surgical radicality [15]. As a result, fibrous adenomas often require very challenging surgical procedures, such as extracapsular dissection [14], as well as second and third-line therapeutic strategies, including second stage surgery, stereotactic radiotherapy or transcranial approaches [16], and are hence associated with a correspondingly higher risk of complications [13]. However, the prognostic impact of adenoma consistency in relation to endocrine complications such as the development of postoperative pituitary deficiencies needs to be explored in detail.

The primary aim of the present study was to assess the impact of pituitary adenoma consistency on postoperative hormone function. Furthermore, we aimed to identify possible predictors of pituitary function, among pre- and post-TSA evaluations. Finally, we sought to investigate whether tumor consistency might be linked with morphological and histological features, as well as worse surgical outcomes.

## Materials and methods

This is an observational, retrospective analysis on patients attending our outpatient Endocrinology clinic who underwent endoscopic TSA in the Neurosurgery Unit of Policlinico Umberto I Hospital, Sapienza University, Rome, Italy, between January 2017 and January 2021.

The study was approved by the ‘Sapienza’ University of Rome Ethics Committee (reference number 6288). Written informed consent was obtained from each patient after full explanation of the purpose and nature of all procedures used. The study was performed in accordance with the principles of the Declaration of Helsinki.

The inclusion criteria were: age > 18 years; diagnosis of pituitary adenoma confirmed by postoperative histology according to the “WHO 2017 classification for tumors of endocrine organs” [17] and treated via surgical resection. The exclusion criteria were: parasellar lesions other than pituitary adenomas (including meningiomas, Rathke’s cleft cysts, metastases, craniopharyngioma, meningiomas); patients who underwent TSA in other hospitals or whose endocrine follow-up was not available up to the 6 months timepoint.

A total of 50 patients who had complete endocrine post-TSA evaluations were identified. Data on age, sex, tumor size, hormone function and tumor histology were collected via a review of medical records.

All patients underwent biochemical evaluation of pituitary function at the following timepoints: preoperative, 3 months postoperative and 6 months postoperative. The difference in pituitary hormone deficiencies between follow-up evaluations and baseline was expressed as Δdeficiency_3M_ (deficiency at 3 months minus deficiencies at baseline) and Δdeficiency_6M_ (deficiencies at 6 months minus deficiencies at baseline). Blood samples were collected at 8 a.m. after 8 h fasting, in the absence of replacement therapy.

Biochemical remission was defined as the normalization of PRL levels for prolactinomas, the presence of normal GH and IGF1 levels (adjusted for sex and age) for acromegaly, and the occurrence of postsurgical hypocortisolemia (morning serum cortisol levels < 50 nmol/L) for Cushing’s disease [18].

### MRI study

All patients underwent preoperative and 6 months postoperative neuroradiological evaluation with contrast-enhanced magnetic resonance imaging (MRI). All MRI images were assessed by an endocrinologist with expertise in neuroradiology. Longitudinal, sagittal and coronal non-enhanced and/or enhanced T1-weighted images, as well as coronal T2-weighted images, were retrieved for all patients. Tumor volume was calculated by the ellipsoid formula (0.52 × a × b × c, with a, b, and c being longitudinal, sagittal, and coronal diameters, respectively), in line with current Literature [19]. Local invasiveness and suprasellar extension were then evaluated. Finally, data on tumor intensity in the T1-weighted and T2-weighted images and enhancement pattern following gadolinium-based contrast administration were collected.

Postoperative MRI studies were used to determine the resection rates following TSA. Specifically, a resection greater than 90% of preoperative volume was considered to be total (gross total resection [GTR]) or near-total resection [NTR]), whereas a resection lower than 90% was considered subtotal (STR).

### Neurosurgical assessment of adenoma consistency

Data on tumor consistency and macroscopic appearance, neurosurgical approach and any intraoperative complications were collected from the surgical records. All pituitary adenomectomies were performed through an endoscopic transsphenoidal approach by a single surgeon (AGR) in the Neurosurgery Ward of Policlinico Umberto I, Sapienza University Hospital. None of the included patients underwent deliberate resection of healthy pituitary tissue during the surgical procedure. The surgical reports were reviewed in order to assess intraoperative consistency; in accordance with the classification previously reported by Bahuleyan et al. [16], suctionable, easily removed lesions were classified as “soft”, whereas firmer, non-suctionable tumors were classified as “fibrous”.

### Tumor histology

All intraoperative tumor samples were examined by our pathology unit. Immunohistochemistry was performed using an appropriate combination of immunostains for the main pituitary hormones (prolactin = PRL, growth hormone = GH, adrenocorticotropic hormone = ACTH, thyroid stimulating hormone = TSH, luteinizing hormone = LH, follicle stimulating hormone = FSH) and the proliferation index (MIB-1/Ki-67 antibody) and p53 expression were also assessed.

### Statistical methods

Distribution of continuous variables was tested with the Shapiro–Wilk test; linearity was established by visual inspection of a scatterplot. Categorical variables are expressed as percentage and frequency; continuous variables are reported as mean and 95% confidence interval (95% CI) or median and interquartile range (IQR, 25th–75th percentile), as appropriate. Bivariate correlations between continuous variables were performed using the Pearson’s correlation coefficient. Comparative analysis between categorical variables was carried out using the χ^2^ test.

*P* values < 0.05 were considered statistically significant. A Kruskal–Wallis test was conducted to determine any differences between groups; distribution between groups was assessed by visual inspection of boxplots. Differences between groups are presented as mean rank differences and standard error (SE). Receiver-operating characteristic (ROC) curve analysis was performed using a uniform threshold for any variables according to the 95% sensitivity on the ROC analysis. Area under the curve (AUC) analysis was used to express the overall diagnostic accuracy of the index criterion.

All statistical analyses were performed using SPSS software Version 24 (IBM).

## Results

### Patient selection

**Fig. 1:**
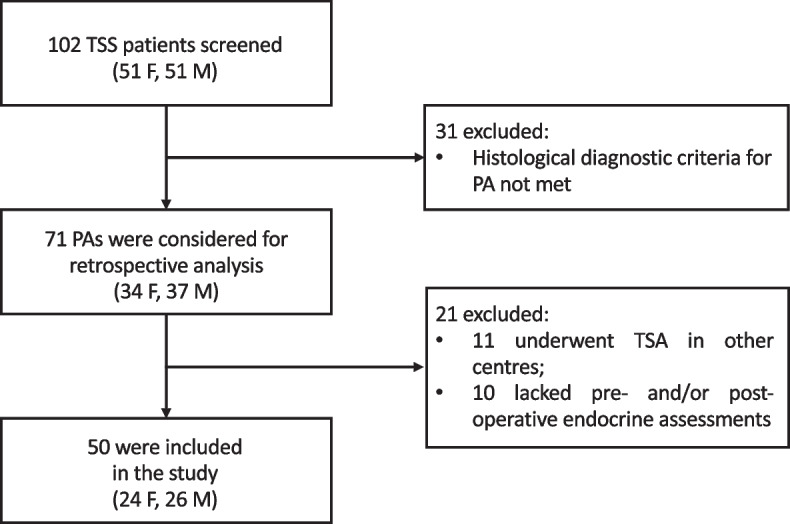
Flow-chart detailing the patient selection process. TSS: transsphenoidal surgery. PA: pituitary adenoma

The patient selection process is summarized in Fig. 1. Over the last 5 years (January 2017- January 2021), 102 patients have attended our pituitary outpatient clinic after endoscopic transsphenoidal surgery for sellar lesions. Of these, 31 were excluded due to the histological confirmation of non-adenomatous lesions, namely Rathke’s cleft cyst (*n* = 11), craniopharyngioma (*n* = 8), meningioma (*n* = 4), germinoma (*n* = 1), chordoma (*n* = 1), astrocytoma (*n* = 1), medulloblastoma (*n* = 2), primitive neuroectodermal tumor (*n* = 1), and pituitary metastasis (*n* = 1). After narrowing our study sample to pituitary adenomas, patients whose TSA was performed in other centers (*n* = 6) were excluded. Lastly, we excluded patients whose hormone panel was not available at the preoperative and/or up to the 6-month postoperative assessments (*n* = 15), leading to the final inclusion of 50 patients who had undergone TSA at the “Sapienza” University of Rome Neurosurgery Unit.

### Patients’ characteristics

The baseline characteristics of the patients are presented in Table 1. The mean age for the whole cohort was 57 ± 13 years; 26 of the 50 patients (52%) were male. Forty-one patients (82%) presented with pituitary macroadenoma. Median tumor volume was 4800 mm^3^ (95% CI 620–8828). Thirty patients (60%) had preoperative visual defects. Both suprasellar extension and cavernous sinus invasion (CSI) were observed in twenty patients (40%). Eight PAs (16%) appeared hyperintense in T2-weighted images, six (12%) were isointense and thirty-six (72%) were hypointense. After contrast administration, twenty lesions (40%) showed homogeneous enhancement. Hormone hypersecretion was observed in 20 cases (40%): specifically, 10 patients (20%) had been previously diagnosed with acromegaly, 7 (14%) with Cushing’s disease, and 3 (6%) presented with prolactinomas. Only three patients (6%) were under anti-secretory and/or anti-proliferative medical treatment before surgery: namely, two patients with prolactinomas (4%) were on dopamine-agonists therapy, whereas one acromegalic patient (2%) was receiving treatment with somatostatin analogs. Thirty-three patients (66%) had preoperative pituitary deficiencies, with 17 (34%) presenting with 2 or more deficiencies. The most common endocrine deficiency was hypogonadism (26 patients, 52%), followed by hypothyroidism (23 patients, 46%) and hypocortisolism (12 patients, 24%), whereas a GH deficiency was observed in a limited number of cases (5 patients, 10%). None of the patients presented with Diabetes Insipidus in the preoperative assessment.

**Table 1:** Baseline characteristics

Parameter	Value
No	50
Age ± SD, years	57 ± 13
Sex, no. (%)	
Male	26 (52%)
Female	24 (48%)
Total tumor volume (mm^3^)	4800.9 (18.72 – 26,087.82)
Visual impairment	28 (56%)
Suprasellar extension	20 (40%)
Cavernous sinus invasion	20 (40%)
No. of impaired pituitary axes per patient	
0	17 (34%)
1	15 (30%)
2	7 (14%)
3	7 (11%)
4	4 (8%)
Tumor consistency (%)	
Soft	45 (90%)
Fibrous	5 (10%)
Tumor histology, n. (%)	
Null cell	9 (18%)
PRL +	6 (12%)
ACTH +	10 (20%)
GH +	14 (28%)
FSH/LH +	15 (30%)

Baseline characteristics of 50 patients undergoing surgical resection for pituitary adenoma

The neurosurgical and histological evaluations are summarized in Table 1. All patients underwent PA resection via endoscopic transsphenoidal surgery. A sellar approach was used in 42 cases (84%), while 8 tumors (16%) required an extended neurosurgical approach. Ultrasound aspiration was performed in 24 cases (48%).

The immunohistochemical assessment revealed the following stratification (Table 1): 15 tumors (30%) displayed cell positivity for FSH/LH, whereas positivity for PRL, GH and ACTH was found in 6 (12%), 14 (28%) and 10 (20%) cases, respectively. In 9 cases (18%), no positivity was observed for any of the pituitary hormones, resulting in a *“non-immunoreactive”* classification. p53 expression was found in 9 cases (18%) and ki67 > 3% in 4 cases (8%). Pituitary apoplexy was observed in 5 patients (10%).

### Preoperative parameters associated with pituitary deficiencies

**Fig. 2:**
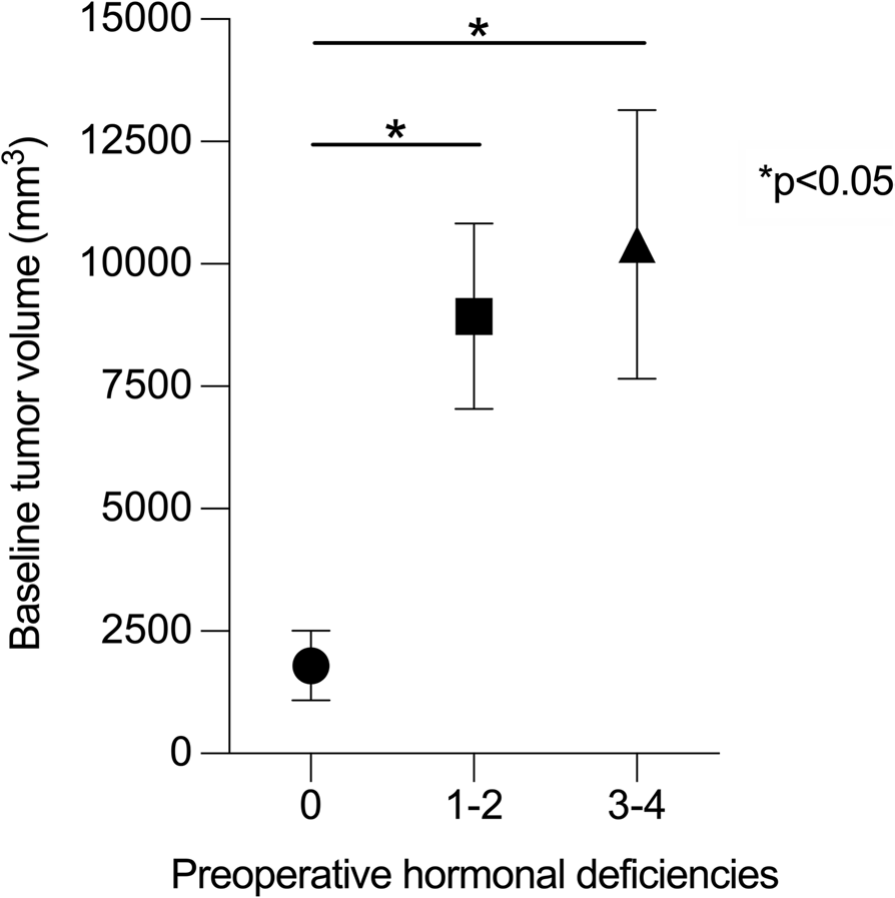
Distribution of preoperative pituitary deficiencies according to baseline tumor volume. Patients were stratified to three subgroups according to the severity of baseline hypopituitarism: absent (0 deficiencies), mild (1–2 deficiencies), severe (3–4 deficiencies)

Tumor volume was directly correlated with the number of hormone deficiencies at baseline (*r* = 0.487, *p* < 0.001). As shown in Fig. 2, when stratified for baseline hormone deficiencies, patients without deficiencies had a smaller tumor than patients with one (-12.625, SE 4.904, *p* = 0.01), two (-15.125 mm^3^, SE 5.951, *p* = 0.01), three (-16.292 mm^3^, SE 6.287, *p* = 0.01) or four (-21.792 mm^3^, SE 8.263, *p* < 0.01) deficiencies (χ^2^ = 14.621, *p* = 0.006).

Furthermore, microadenomas tended to be more frequent in women (χ^2^ = 3.706, *p* = 0.07; OR 4.813, 95% CI 0.881–26.299), who were also more likely to display normal preoperative pituitary function (χ^2=^12.178, *p* < 0.001; OR 10.733, 95% CI 2.5–45.813) compared to men.

**Fig. 3:**
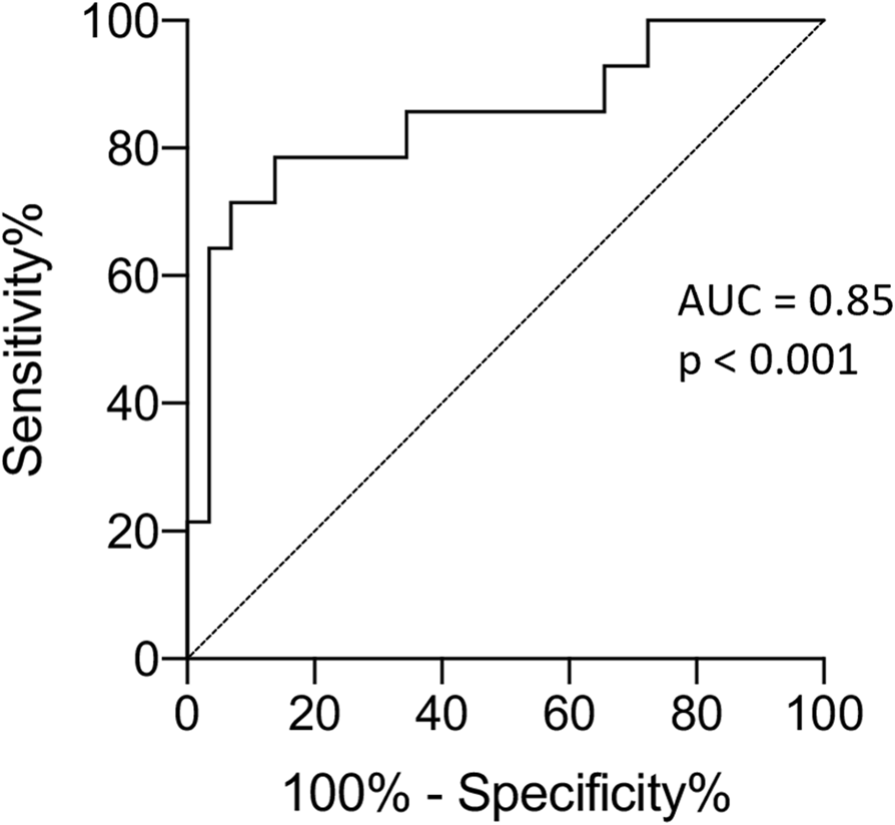
ROC curve analysis assessing the ability of baseline tumor volume to predict the presence or absence of preoperative hormone deficiencies. A threshold of 620 mm^3^ effectively predicted preoperative pituitary function (AUC = 0.85, sensitivity 95%, specificity 66%)

We then tried to determine possible predictors for preoperative endocrine deficiencies from the dimensional information gained from the MRI analysis. According to the ROC analysis (Fig. 3), preoperative smaller tumor volume best predicted the absence of pituitary deficiencies (AUC = 0.85; *p* < 0.001), even when compared to the conventionally measured diameters: cranio-caudal diameter (CCD, AUC = 0.84, *p* < 0.001); latero-lateral diameter (LLD, AUC = 0.78; *p* = 0.002); and antero-posterior diameter (APD, AUC = 0.84; *p* < 0.001). Moreover, a volume greater than 620 mm^3^ was identified as a cut-off for predicting an increased likelihood of any preoperative pituitary deficiency (95% sensitivity, 66% specificity). When considering a cut-off of 95% sensitivity for all variables, tumor volume showed higher specificity (66%) than single diameters (50% for all three diameters). Age was not associated with preoperative deficiency.

### Tumor consistency and postoperative pituitary deficiencies

Following TSA, all patients were prescribed with 20 mg (or its equivalent) of hydrocortisone replacement therapy following our local post-TSA protocol. Postoperative diabetes insipidus was observed in 12 patients (24%) and was found to be permanent in 6 cases (12%).

All patients underwent postoperative endocrine assessments at 3 and 6 months. The cumulative percentages of hormone deficiencies in our cohort are shown in Table 2.

**Table 2:** Pituitary hormone deficiencies following TSA

	**Baseline**	**3-month follow-up**	**6-month follow-up**
ACTH deficiency	12 (24%)	27 (54%)	27 (54%)
TSH deficiency	23 (46%)	20 (40%)	20 (40%)
FSH/LH deficiency	26 (52%)	21 (42%)	20 (40%)
GH deficiency	5 (10%)	7 (14%)	7 (14%)
Diabetes Insipidus	0 (0%)	6 (12%)	6 (12%)

Percentages describing the distribution of postoperative hormone deficiencies at the 3- and 6-month timepoints

At the 3-month assessment, 27 patients (54%) presented with adrenal insufficiency. Central hypothyroidism, hypogonadotropic hypogonadism and GH deficiency were observed in 20 (40%), 21 (42%) and 7 (14%) patients, respectively. Postoperative diabetes insipidus was found in 6 patients (12%).

At the 6-month assessment, none of the patients with postoperative ACTH deficiency had recovered HPA function. Similarly, there were no changes in the rates of GH and TSH deficiency or diabetes insipidus compared to the 3-month assessment. Furthermore, only one (4%) of the 21 patients with postoperative central hypogonadism had recovered gonadal function at the 6-month assessment.

As shown in Table 2, no differences were found in hormone deficiencies between the 3 and 6 months assessments (*p* > 0.05 for all axes), indicating that hormone deficiencies present at 3 months persisted at the 6-month timepoint. Thus, we will only present data for the 6-month (postoperative) follow-up.

We then aimed to ascertain whether tumor consistency was associated with the development of new pituitary deficiencies. A soft consistency was observed in 45 PAs (90%), with 5 (10%) classified as “fibrous”.

The clinical, radiological and anathomo-pathological characteristics of patients presenting with fibrous tumors are summarized in the Supplementary Table 1.

A comparison of intraoperative consistency with postoperative hormone function showed significant correlations between fibrous consistency and the Δdeficiency at the postoperative evaluation (*r* = 0.313, *p* = 0.02).

**Fig. 4:**
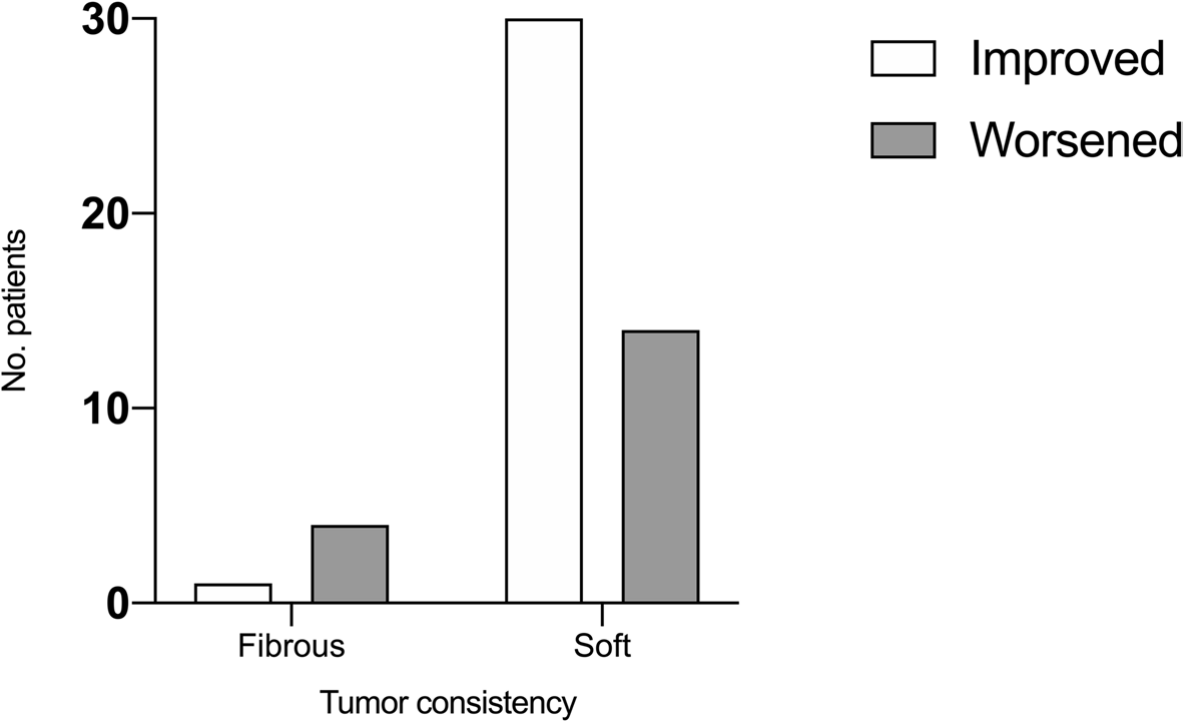
A comparison of postoperative pituitary function between patients with fibrous (left) and soft (right) pituitary adenomas. Hormone function was regarded as improved in the case of a Δdeficiency (postoperative deficiencies minus baseline deficiencies) ≤ 0, and worsened with a Δdeficiency > 0. (*χ^2^ = 4.485, *p* = 0.05)

To further test the strength of this association, the cohort was stratified according to the development of postoperative pituitary deficiencies. Patients who presented a Δdeficiency less than or equal to 0 (indicating improved or stable pituitary deficiencies compared to baseline) were compared against patients who exhibited a Δdeficiency greater than 0 (reflecting the development of new pituitary deficiencies). Of the patients with fibrous tumors, 80% presented a Δdeficiency > 0 compared with 32% of patients with soft adenomas (χ^2^ = 4.485, *p* = 0.05, Fig. 4), thus demonstrating that the former had a higher risk of developing new postoperative deficiencies (OR, 8.571; 95% CI, 0.876 to 83.908).

### Tumor consistency and preoperative parameters

To identify possible predictors of tumor consistency, we investigated whether a fibrous texture was associated with any preoperative features, both radiological and clinical. As shown in Supplemental Table 1, three patients with fibrous adenomas presented with functioning tumors (two prolactinomas, one GH-secreting adenoma), whereas the remaining two (40%) exhibited non-functioning tumors. None of the patients with fibrous, functioning tumors received medical treatment prior to surgery. All fibrous tumors were macroadenomas, with three of them (60%) exhibiting radiological signs of local invasiveness. Our data did not highlight any significant correlations with preoperative parameters, including tumor intensity in T1- and T2-weighted images, post-contrast enhancement pattern, CSI, optic chiasm compression, hormonal hypersecretion, and tumor subtype.

### Tumor consistency and postoperative biochemical remission

We then tried to determine whether tumor consistency could affect the chance of achieving post-surgical, biochemical remission in functioning adenomas. Among the 20 patients with functioning tumors, 17 presented with soft adenomas and 3 with fibrous tumors. Namely, 8 patients in the “soft” group achieved biochemical remission following surgery, whereas no patient in the “fibrous” group did; this difference was not significant (χ2 = 2.353, *p* = 0.125).

### Tumor consistency and clinical aggressiveness parameters

**Fig. 5:**
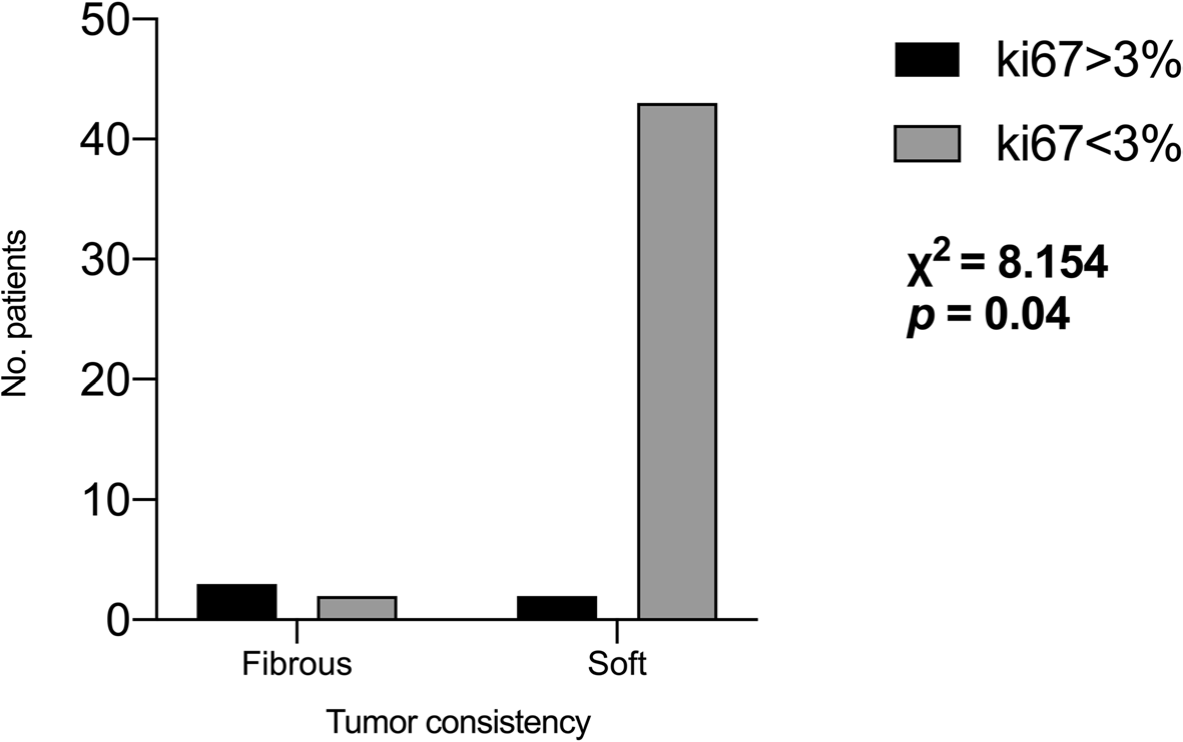
A comparison of Ki-67 (MIB-1) staining index expression between patients with fibrous (left) and soft (right) pituitary adenomas. A cut-off of 3% was considered suggestive of potentially aggressive clinical behavior. (*χ^2^ 8.154, *p* = 0.04)

We then examined possible relationships between adenoma consistency and other important variables commonly associated with clinical aggressiveness, including ki67% and p53 expression, cavernous sinus invasion and tumor volume. Fibrous consistency was not associated with p53 overexpression, cavernous sinus invasion or tumor volume. However, although only 5 patients presented fibrous tumors, 3 of these (60%) expressed ki67 > 3%, compared to only 5% in the “soft” subgroup (χ^2^ 8.154, *p* = 0.04, Fig. 5), resulting in a 17-times higher possibility of expressing high ki67 levels (OR 17.500, 95% CI 1.551–197.435).

### Parameters associated with surgical radicality

Median tumor volume following pituitary surgery was 28 mm^3^ (95% CI 0–5779.8). GTR was achieved in 32 cases (64%), with a complete surgical resection in 23 patients (46%). Age, T1- and T2-weighted tumor intensity, contrast enhancement and baseline tumor volume were not associated with impaired radicality. Similarly, functional status did not affect surgical radicality (χ2 = 2.037, *p* = 0.153) nor the development of additional postoperative deficiencies (χ2 = 0.045, *p* = 0.833), and resection rates were similar between functioning and non-functioning tumors (*p* = 0.168). Conversely, worse resection rates were observed in tumors with suprasellar extension (χ2 = 5.048, *p* = 0.02) and CSI (χ^2^ = 4.000, *p* = 0.04), resulting in a sixfold (OR 6.000, 95% CI 1.129–31.880) and threefold (OR 3.857, 95% CI 0.997–14.916) increase of risk of lower surgical radicality, respectively. Similarly, our analysis demonstrated that tumor consistency affects surgical radicality, as a fibrous consistency was significantly associated with a lower resection rate (χ2 = 8.148, *p* = 0.004; OR 1.385, 95% CI 1.040–1.844).

## Discussion

Our study aimed to explore the relationship between tumor consistency and postoperative endocrine function in patients with pituitary adenoma undergoing TSA, demonstrating that increased adenoma consistency is associated with the development of new-onset postoperative pituitary deficiencies. Moreover, our study confirmed pituitary adenoma volume as a reliable predictor for preoperative hormone deficiencies, as well as the sex differences in clinical features of patients affected by pituitary adenoma. Hypopituitarism, defined as a loss of hormone secretory function which can affect one or more pituitary axes, is a rare condition, with an estimated prevalence of 290 to 455 cases per million in the general population [20], and is associated with an increase in overall mortality [21] and a decrease in quality of life [22]. In adults, hypopituitarism is generally associated with the presence of intrasellar lesions – usually pituitary adenomas [23]—exerting a compressive effect on healthy pituitary parenchyma. Several predictors of preoperative endocrine dysfunction have been identified, including older age, male sex, and tumor size [24]. Although no association was found with age, our results were consistent with these findings, as male sex and baseline tumor volume were found to be strongly associated with preoperative dysfunction. In keeping with previous data [24], our study also highlighted a differential susceptibility to compression among hormone-secreting cells, with gonadotroph and thyrotroph cells being the most affected (in 52% and 46% of our patients, respectively).

The micro/macroadenoma distinction traditionally plays a prominent role in the clinical approach to patients with pituitary adenomas [25]. Maximum diameter is currently regarded as the best clinical indicator for evaluating tumor size in a wide variety of neoplasms, including pituitary adenomas [26]; conversely, the assessment of tumor volume is seldom employed in clinical settings and is more commonly used in an experimental context [27]. However, previous studies have demonstrated that maximum dimension is often ineffective in predicting the clinical impact of pituitary adenomas, especially since it can lead to a gross underestimation of tumor growth, as small variations in tumor diameters have a cuboidal relationship with changes in tumor volume [28]. In line with these findings, our data demonstrated that tumor volume, computed via the simple ellipsoid formula (see methods), can be an even better predictor of preoperative hormone dysfunction, with higher specificity when compared against tumor diameters. Therefore, it can be regarded as a helpful tool in assessing the clinical significance of pituitary adenomas, with good accuracy and low inter-rater variability, although its usefulness could be limited in selected cases, such as giant and/or irregularly shaped tumors [25].

Aside from the mass effect-related damage on the healthy pituitary gland, anterior pituitary dysfunction can also derive from iatrogenic injuries involving the sellar region and is generally regarded as one of the most frequent complications of pituitary surgery, occurring in 5–25% of cases [29–31]. Following surgical manipulation, postoperative hormone function largely depends on the state of healthy gland tissue. Hormone secretion can be preserved, or even improved, if enough healthy pituitary tissue is spared; conversely, the destruction of gland parenchyma generally results in the need for lifelong hormone replacement therapy [32, 33]. The endoscopic transsphenoidal approach has now replaced the microscopic technique as the preferred method for the resection of pituitary lesions, due to the improved visualization of critical neurovascular structures and the operating field as a whole, resulting in a lower chance of iatrogenic complications [34]. Several endoscopic series have documented great variability regarding postoperative improvement in pituitary function, ranging from 21 to 63% of cases [7, 10, 35, 36]. Furthermore, a recent meta-analysis of 38 studies found no significant difference in the rate of postoperative hypopituitarism between the endoscopic and microscopic transsphenoidal approach [37]. Of note, a new exoscope-based surgical approach has emerged as a promising alternative for pituitary surgeries [38]; however, data concerning its impact on long-term complications, including postoperative hypopituitarism, are still scarce [39], further underscoring that endocrine outcomes following pituitary surgery are largely unpredictable [24].

In our study, 19 patients (38%) showed worse postoperative pituitary function compared to baseline while 10 patients (20%) had improved, a lower percentage than observed in most series [15, 35]. The low recovery rate observed in our cohort is likely attributable to the short duration of the observation period, as several studies have reported endocrine recovery several months or even years after surgery [24, 40]. Moreover, our retrospective analysis was carried out in a real-life setting and therefore also included large, invasive tumors, some of which proved particularly challenging from a neurosurgical standpoint: this may also be a factor in the lower postoperative hormone recovery rate.

Furthermore, to provide a “real-life” perspective, consecutive pituitary tumors were included in the study, regardless of their functional status. Notably, functioning tumors can be subjected to a higher resection rate due to a more aggressive neurosurgical approach to achieve biochemical remission; this can also result in a potentially higher rate of postoperative deficiencies due to a more extensive manipulation of healthy pituitary tissue. However, in our cohort, no significant differences were observed regarding neither resection rates or the incidence of additional postoperative deficiencies between functioning and non-functioning tumors, ruling out possible confounding effect of tumor functional status. Moreover, in some functioning tumors – especially ACTH-secreting adenomas – prolonged hormone deficiency is generally a desirable outcome, as it predicts long-term remission [41]; nevertheless, ACTH deficiencies in patients previously affected by Cushing’s disease were still included in the count of postoperative pituitary deficits.

A wide variety of factors has been implicated in the onset of postoperative hypopituitarism, including the surgeon’s expertise [42] and the surgical approach [29], as well as intrinsic characteristics of the adenoma, such as tumor volume [43, 44] and invasion of the adjacent anatomical loci (primarily the cavernous sinuses) [10, 32].

Tumor consistency has recently emerged as a critical factor in surgical planning for a wide variety of brain neoplasms, influencing both the surgical outcome and the onset of postoperative complications [14, 45].

Pituitary adenomas are generally soft, but approximately 5–13% present a firm consistency in the intraoperative assessment [11, 46]. Softer adenomas are easier to remove and are generally associated with easy suction and a lower prevalence of intraoperative complications. In contrast, a firmer consistency is associated with increased intratumoral collagen content and higher cellularity [47], and has been linked to radiological features traditionally associated with unfavorable clinical outcomes, including high-degree cavernous sinus invasion [48], suprasellar extension and/or dumbbell appearance [49], and intratumoral hemorrhage [50]. Along these lines, our results support the hypothesis that fibrous tumors might be endowed with increased tumoral proliferation, as fibrous consistency was found to be associated with an increased likelihood of a high ki67 index during the histological assessment. No prospective studies have yet analyzed the relationship between pituitary tumor consistency and histological markers of aggressiveness. While further confirmation with a larger sample is required, our findings are in agreement with previous studies linking fibrous texture with increased cellularity and a high nucleus-to-cytoplasm ratio [47, 51]. Moreover, a firmer consistency has been demonstrated to considerably impair surgical removal by hindering both the debulking and the mobilization of pituitary tumors [52]. As a result, fibrous adenomas were previously considered to be only resectable via a transcranial route, in light of the difficulty in achieving adequate debulking via suction and/or curettage with a standard transsphenoidal approach [53]. However, the advent of the expanded endonasal endoscopic approach has considerably widened the indications for transsphenoidal surgery to include dumbbell-shaped tumors and fibrous adenomas [49], although careful preoperative planning is required [11, 54].

To our knowledge, despite current evidence linking tumor consistency to the surgical outcomes for pituitary adenomas [14], its impact on postoperative endocrine function is still largely unexplored, with few published series recently suggesting an association with higher rates of postoperative hypopituitarism [55–57]. In accordance with current Literature, 10% of tumors in our cohort presented as fibrous during the intraoperative assessment. Interestingly, tumor consistency influenced the development of new postoperative endocrine deficiencies, as fibrous tumors were associated with a greater Δdeficiency at the postoperative assessment, as well as an 8-times higher risk of developing new hormone deficiencies compared to soft adenomas.

These findings can likely be ascribed to the firm texture that might preclude surgical radicality, especially in light of recent evidence citing tumor consistency as one of the main limiting factors in the removal of pituitary adenomas [11, 54]—even more so than preoperative tumor volume [14, 49]. Indeed, our data showed that adenoma consistency, and not baseline tumor volume, can greatly affect surgical radicality, along with the presence of CSI and suprasellar extension. In keeping with our findings, several studies have reported that increased adenoma consistency is correlated with incomplete tumor resection in patients undergoing TSA [58], as exemplified by lower gross total resection rates (GTR) [15] compared to soft adenomas, in which surgical radicality is more frequently achieved [11, 54]. Incomplete tumor resection has been demonstrated to greatly affect several clinical outcomes and has been associated with higher rates of recurrence: up to 46% of cases, compared to the 12% observed in the absence of visible tumor residues [59]. Furthermore, is it widely acknowledged that failure to achieve surgical radicality results in an overall poorer prognosis [60] and a higher rate of postoperative pituitary deficiencies [32, 61]. Pituitary surgery should thus aim for maximum radicality. However, firm adenomas have proven difficult to mobilize properly, especially in the event of suprasellar and parasellar extension. In such instances, more complex surgical strategies are required, such as craniotomy or a two-stage transsphenoidal operation, to reduce the risk of damaging healthy pituitary tissue and/or the arachnoid membrane [12]. Alternatively, in order to achieve a more radical removal in a single surgical setting, a widened surgical approach is often warranted [62]. The higher chance of developing new hormone deficiencies in fibrous adenomas is likely also related to the lengthier, more complex neurosurgical maneuvers, which involve a higher risk of damaging healthy pituitary tissue. Therefore, knowing the tumor’s consistency before surgery might thus prove useful not only in surgical planning, but also in optimizing endocrine follow-up. To date, the accurate prediction of pituitary adenoma consistency via preoperative MRI is still under debate [63]. Promising evidence has suggested that fibrous consistency may be predicted on the basis of preoperative morphological features assessed via contrast-enhanced pituitary MRI. Increased collagen content has been associated with lower intensity in T2-weighted images [47, 64], with a lower apparent diffusion coefficient (ADC) in diffusion-weighted imaging [65], and with higher elastography values [66]. Nevertheless, our analysis did not highlight any significant association between adenoma consistency and preoperative tumor morphology, likely due to the relatively small sample size. Today’s standard MRI protocols for pituitary adenomas include pre-contrast T1- and T2-weighted coronal and sagittal scans as well as post-contrast coronal scans. As diffusion-weighted studies are not routinely performed [67], the diagnosis of fibrous consistency is often intraoperative. In our view, the discovery of a firm lesion should prompt a more dedicated clinical follow-up, especially given the increased risk of developing postoperative hormone deficiencies.

Current guidelines recommend retesting for all pituitary axes 6 weeks after pituitary surgery, but the timing for follow-up evaluations is less clearly defined [68]. Even if this was not the aim of our study, we found a strong correlation between postoperative deficiencies at the 3- and 6-month assessments, suggesting an overall persistence and a lack of functional recovery between the two consecutive evaluations. This result thus suggests that, in most cases, pituitary axes should be serially re-evaluated at 6 weeks and subsequently at 6 months after surgery. Particular attention should be paid to patients identified by the neurosurgeon as affected by fibrous adenoma, because of the high risk of pituitary deficiency. The multidisciplinary approach, through a strict collaboration between endocrinologist and neurosurgeon, is therefore essential for the correct management of the patients with pituitary adenoma.

The main limitations of this study are its retrospective nature and the relatively small sample size. Furthermore, the lower proportion of patients with pre- and postoperative GH deficiency in our series may result from a testing bias, as dynamic GH stimulation tests can prove difficult to administer and measure. For this reason, GH deficiency was diagnosed according to IGF-1 levels, and it was assumed in the concomitant presence of at least three other pituitary deficiencies, in accordance with current guidelines [68]. Moreover, tumors found to be negative for hormonal immunostainings were classified as “non-immunoreactive” adenomas due to the lack of routinary assessment of transcription factors at the time of surgery. Lastly, these results—despite their accordance with current literature—should be interpreted in the context of the small sample of fibrous adenomas, and should therefore be regarded as preliminary, albeit promising, findings shedding light on the possible role of tumor consistency in postoperative pituitary function.

In conclusion, our study suggests that increased tumor consistency is associated with the development of postoperative pituitary deficiencies. The gold standard for defining pituitary adenoma consistency is currently postoperative histological assessment, which can provide reliable information concerning the lesion’s collagen content, as well as its cellularity [47, 51, 65]. However, the evaluation of tumor consistency is not routinely included in diagnostic protocols concerning pituitary lesions; as a result, fibrous adenomas are generally diagnosed by neurosurgeons, during the intraoperative assessment. We therefore believe that our findings further underscore the need for a concerted effort in managing pituitary lesions; more specifically, the intraoperative finding of fibrous pituitary adenoma should prompt a multidisciplinary discussion between the neurosurgeon and the endocrinologist, enabling a more personalized postoperative endocrine management strategy to be planned. Further prospective studies with larger cohorts are needed to better elucidate the role of pituitary texture as a potential predictor of postoperative complications, including endocrine dysfunction.

## Supplementary Information

Additional file 1: Supplementary Table 1. Baseline and post-surgical characteristics of patients with fibrous pituitary adenomas.As per journal requirements, every additional file must have a corresponding caption. In this regard, please be informed that the caption was taken from the additional e-file itself. Please advise if the action taken is appropriate and amend if necessary.I confirm that the caption is appropriate.

## Data Availability

The datasets used and/or analyzed in the present study are available from the corresponding author on reasonable request.

## Abbreviations

PA: Pituitary adenoma
TSA: Trans-sphenoidal adenomectomy
MRI: Magnetic resonance imaging
GTR: Gross total resection
NTR: Near total resection
STR: Sub-total resection
PRL: Prolactin
GH: Growth hormone
ACTH: Adrenocorticotropic hormone
TSH: Thyroid stimulating hormone
LH: Luteinizing hormone
FSH: Follicle stimulating hormone
ROC: Receiver operating characteristic
AUC: Area under the curve
