# Supplementary material for: Pituitary adenoma consistency affects postoperative hormone function: a retrospective study
Source: BMC Endocr Disord. 2023 Apr 26;23:92. doi: 10.1186/s12902-023-01334-1 (PMC10131333; doi:10.1186/s12902-023-01334-1)
Supplement: Supplementary file 1 — Additional file 1: Supplementary Table 1. Baseline and post-surgical characteristics of patients with fibrous pituitary adenomas.As per journal requirements, every additional file must have a corresponding caption. In this regard, please be informed that the caption was taken from the additional e-file itself. Please advise if the action taken is appropriate and amend if necessary.I confirm that the caption is appropriate. [file 12902_2023_1334_MOESM1_ESM.docx]

| **Patient n.** | **Sex** | **Age** | **Tumor Subtype** | **Extrasellar involvement** | **MRI features** | **Tumor volume (mm^3^)** | **Pre-surgical medical treatment** | **Volume reduction post-surgery (%)** | **Pre-surgical hormonal deficiencies** | **Post-surgical hormonal deficiencies** | **Biochemical remission** |
| --- | --- | --- | --- | --- | --- | --- | --- | --- | --- | --- | --- |
|  |  |  |  |  |  |  |  |  |  |  |  |
| #1 | M | 40 | GH^+^, functioning | cavernous sinus, optic chiasm | iso in T1w and T2w images | 29047.20 | no | 37.0 | ACTH, FSH/LH, TSH | ACTH, FSH/LH, TSH, ADH | no |
|  |  |  |  |  |  |  |  |  |  |  |  |
| #2 | F | 67 | PRL^+^, functioning | cavernous sinus, optic chiasm | hypo in T1w and T2w images | 2592.84 | no | 40.1 | FSH/LH | FSH/LH, TSH, ADH | no |
|  |  |  |  |  |  |  |  |  |  |  |  |
| #3 | M | 49 | ACTH^+^, nonfunctioning | optic chiasm | iso in T1w and T2w images | 5166.72 | NA | 90.8 | FSH/LH | FSH/LH, GH, TSH | NA |
|  |  |  |  |  |  |  |  |  |  |  |  |
| #4 | F | 58 | PRL^+^, functioning | optic chiasm | - | - | no | - | FSH/LH | ACTH, FSH/LH, GH, TSH | no |
|  |  |  |  |  |  |  |  |  |  |  |  |
| #5 | F | 77 | FSH^+^, nonfunctioning | cavernous sinus, optic chiasm | iso in T1w and T2w images | 19522.88 | NA | 69.3 | TSH | ACTH, TSH | NA |
|  |  |  |  |  |  |  |  |  |  |  |  |

**Supplementary Table 1. Baseline and post-surgical characteristics of patients with fibrous pituitary adenomas.**

Clinical, radiological and histological features of patients presenting with fibrous pituitary adenomas. ACTH = Adrenocorticotropic hormone; ADH = Anti-diuretic hormone; FSH = Follicle stimulating hormone; GH = Growth hormone; LH = Luteinizing hormone; PRL = Prolactin; TSH = Thyroid stimulating hormone; Iso: isointense; Hypo: hypointense; T1w: T1-weighted; T2w: T2-weighted; NA: not applicable
